# Upper Bounds for Chebyshev Permutation Arrays

**DOI:** 10.3390/e27060558

**Published:** 2025-05-26

**Authors:** Sergey Bereg, Zevi Miller, Ivan Hal Sudborough

**Affiliations:** 1 Department of Computer Science, University of Texas at Dallas, Box 830688, Richardson, TX 75083, USA; hal@utdallas.edu; 2 Department of Mathematics, Miami University, Oxford, OH 45056, USA; millerz@miamioh.edu

**Keywords:** permutation arrays, Chebyshev metric, upper bounds

## Abstract

We improve on known upper bounds for the size of permutation arrays under the Chebyshev metric, defined as follows. The Chebyshev distance between permutations π and σ on the symbols {1,2,…,n}, denoted by d(π,σ), is $\max\{|\pi_{i}-\sigma_{i}|\;|\;1\leq i\leq n\}$. For an array *A* (set) of such permutations, the Chebyshev distance of *A*, denoted by d(A), is min{d(π,σ)|π,σ∈A,π≠σ}. An array *A* of such permutations with d(A)=d will be called an (n,d)-PA. Let P(n,d) denote the maximum size of any (n,d)-PA. The function P(n,d) has been the subject of previous research. In this paper, we consider strings on the symbols {0,1,2}, with the 0’s representing low symbols and the 2’s high symbols for the function P(n,d). An array *A* of such strings of length *n* is *separable* if for any two strings in *A*, there is a position 1≤i≤n such that the $i^{th}$ symbol in one string is 0 and the $i^{th}$ symbol in the other is a 2. The maximum size of a separable array of strings of length *n*, with *a* occurrences of the symbol 0 and *b* occurrences of the symbol 2, is denoted by R(n;a,b). We show that R(n;k,k) is an upper bound for P(n,n−k) when $k\leq \frac{n}{2}$. We derive upper bounds for R(n;a,b) by various recursive and combinatorial methods, from which follow upper bounds for the Chebyshev function P(n,d), which improve upon previous such upper bounds in the literature.

## 1. Introduction

In refs. [1,2], studies of permutation arrays under the Chebyshev metric were presented. This complemented many studies of permutation arrays under other metrics, such as the Hamming metric [3,4,5], Kendall τ metric [6,7], and several others [8]. The use of the Chebyshev metric was motivated by applications of error correcting codes and recharging in flash memories [6].

The flash memory application is based on a rank-modulation scheme [9], which eliminates the need to use absolute values of cell levels in storing information. Instead, relative ranks are used. The data are coded by permutations of a finite number of ranks.

Let $\pi =\pi_{1}\pi_{2}\ldots \pi_{n}$ and $\sigma =\sigma_{1}\sigma_{2}\ldots \sigma_{n}$ be two permutations on the symbols in {1,2,…n}. The Chebyshev distance between π and σ, denoted by d(π,σ), is $\max\{|\pi_{i}-\sigma_{i}|\;|\;1\leq i\leq n\}$. For an array (set) of permutations, say, *A*, the Chebyshev distance of *A*, denoted by d(A), is min{d(π,σ)|π,σ∈A,π≠σ}. An array *A* of permutations on {1,2,…n} with d(A)=d will be called an *(n,d)-PA*. Let P(n,d) denote the maximum size of any (n,d)-PA. We shall also define analogously the Chebyshev distance between two strings and the Chebyshev distance of an array of strings. The context will make clear whether the objects are strings or permutations.

Previous work on permutation arrays under the Chebyshev metric gave upper bounds based on a Gilbert–Varshamov inequality [10,11] (see our Theorem 2, or, for a recursive inequality, see our Theorem 3). In [2], it was also shown for fixed r≥1 that there exist constants $c_{r}$ and $d_{r}$ such that $P\left(d+r,d\right)=c_{r}$ for $d\geq d_{r}$ (see our Theorem 1). Upper bounds on $c_{r}$ and $d_{r}$ were given in [2]. We give substantial improvements on these upper bounds.

**Figure 1:**
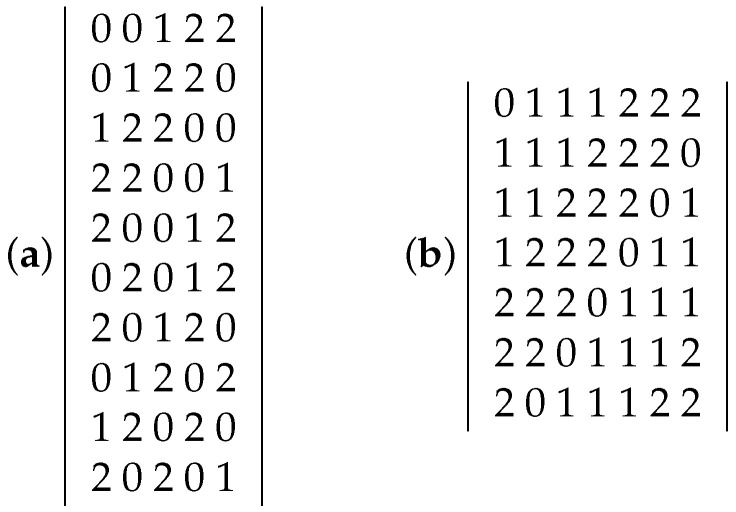
(**a**) (5,2,2)-array with 10 rows and (**b**) (7,1,3)-array with 7 rows.

We consider strings over the alphabet {0,1,2}. A set *A* of such strings of length *n* is *separable* if for any two strings in *A*, there is a position 1≤j≤n such that the $j^{th}$ symbol in one string is 0 and the $j^{th}$ symbol in the other is a 2. We will often view such a set *A* as a matrix in which the rows are the strings in *A* (ordered arbitrarily) and the columns are the coordinate positions 1,2,…,n of entries in these strings. So, the (i,j)’th entry of *A* in this view is the entry (0,1, or 2 ) in the *j*’th position of string *i* of *A*. If every string in a separable array *A* has length *n* and has *a* occurrences of the symbol 0 and *b* occurrences of the symbol 2, then we call *A* an *(n,a,b)-array*. The maximum number of strings in an (n,a,b)-array is denoted by R(n;a,b). Examples of a (5,2,2) array and a (7,1,3) array are shown in Figure 1.

Such matrices, consisting of the three symbols 0, 1, and 2, are reminiscent of weighing matrices. A weighingmatrix *W* of weight *w* is a square matrix of rank *n* containing symbols −1, 0, and 1 such that $W\cdot W^{T}=wI_{n}$ [12]. A weighing matrix is an extension of Hadamard matrices [13] by adding the symbol 0 (see [14]). A circulantweighingmatrix is a weighing matrix in which each row is a circular shift of the first row [14]. There are examples of n×n weighing matrices that can be transformed to an (n,a,b)-array, for appropriate *a* and *b*, by transforming the three symbols (−1, 0, 1) to (0, 1, 2), respectively. For example, the circulant weighing matrix with first row (−1 1 0 1 1 0) is transformed into a circulant (6, 1, 3)-array with first row (0 2 1 2 2 1) by the indicated replacement of symbols.

The motivation for our study begins with Lemma 1, given in the next section, showing that any upper bound for R(n;k,k) also serves as an upper bound for the Chebyshev function P(n,n−k). The resulting upper bounds we obtain on the Chebyshev function give improvements over what was previously known in the literature.

Our results are the following.

1. By means of a transformation, we adapt a result of Bollobás from the theory of extremal sets to show that R(n;s,t)≤2s+2ts+t. From this, we derive P(d+r,d)≤4r2r=24r2πr(1+o(1)) (as *r* grows). This improves on the previously known upper bound for P(d+r,d) for large *r*.
2. We develop recursive methods for upper bounding R(n;s,t). For small *r*, these yield upper bounds for P(d+r,d), which improve on both the previously known upper bounds for P(d+r,d) and on the bound obtained through the transformation of the Bollobás result stated above.

We will need the following notation and terms. For an array *A*, we let |A| be the number of rows in *A*. For any row *r* in an array *A*, we let r(i) be the entry of *r* in column *i* (which we also call *position i*) of *A*. Given a set *S* of rows in *A*, we say that *S* is *separated in a set of columns C* if for any two rows r,s∈S there is a column i∈C such that one of {r(i),s(i)} is 0 while the other is 2. Given two sets $S_{1},S_{2}$ of rows in *A*, we refer to *internal* separations of $S_{1}$ and $S_{2}$ as that set of columns at which either $S_{1}$ is separated or $S_{2}$ is separated. We refer to *cross* separations of $S_{1}$ and $S_{2}$ as that set of columns *C* such that for any two rows $r\in S_{1}\setminus S_{2}$ and $s\in S_{2}\setminus S_{1}$, there is a column c∈C at which {r,s} are separated.

## 2. Background and Preliminary Results

We begin with a theorem by Klove et al. [2], preceded by a definition.

**Definition 1.**  *If A is a (d+r,d)-PA, then the integers 1,2,…,r and d+1,d+2,…,d+r are called potent symbols. Moreover, the integers 1,2,…r are called low potent symbols and the integers d+1,d+2…d+r are called high potent symbols.*

Their proof of the following upper bound, here omitted, uses the idea of potent symbols.

**Theorem 1**  (Klove et al. [2])**.** *For fixed r≥1, there exist constants $c_{r}$ and $d_{r}$ such that $P\left(d+r,d\right)=c_{r}$ for $d\geq d_{r}$. Moreover,*

(1) $$c_{r}\leq 2^{2r}\left(2r\right)!$$

*and*

(2) $$d_{r}\leq 1+\left(2r-1\right)c_{r}-r.$$

Exact values are known for r=1 and r=2, namely, $c_{1}=3$, $d_{1}=2$ [2], $c_{2}=10$, and $d_{2}=3$ [1]. The upper bounds for $c_{r}$ and $d_{r}$ given in (1) and (2) turn out to be quite generous. For example, inequality (1) gives the bound $c_{2}\leq 384$, but we know that $c_{2}=10$. We will see additional improvements on $c_{r}$ as given in Equation (1) later in this paper.

The role of potent symbols motivates the idea behind the following lemma, which establishes the connection between the Chebyshev function and R(n;k,k). Consider an (n,n−k)-PA, which we call *A*, and any row π of *A*. The idea is to put the symbols 1,2,…,n of π into three groups. Those that are high (resp. low) potent symbols, namely, n−k+1,n−k+2,…,n (resp. 1,2,…,k), are relabeled 2 (resp. 0), while all other symbols are relabeled 1. Repeating this replacement over all rows of *A*, independently in any two rows, will yield an (n,k,k)-array, as we will see below. On the other hand, given an (n,k,k) array *B*, we perform the inverse replacement independently in each row of *B* to obtain an (n,n−2k+1)-PA. We obtain bounds linking the *R* function with the Chebyshev function in the lemma following.

**Lemma 1.**  *P(n,n−k)≤R(n;k,k)≤P(n,n−2k+1) when $k\leq \frac{n}{2}$.*

**Proof.**  We begin with the first inequality. Let *A* be an (n,n−k)-PA, where $k\leq \frac{n}{2}$. Create an (n,k,k)-array $A^{\prime }$ as follows. For each row π∈A, create a row $\pi^{\prime }\in A^{\prime }$ by

$$\pi^{\prime }\left(i\right)=\left\{\begin{array}{cc} 0 & \mathrm{if}\;\pi (i)\in \{1,\ldots ,k\},\\ 2 & \mathrm{if}\;\pi (i)\in \{n-k+1,\ldots ,n\},\;and\\ 1 & \mathrm{otherwise}. \end{array}\right.$$

Then, $A^{\prime }$ is an array over the symbols {0,1,2}, having *k* many 0’s and *k* many 2’s in each row. Since d(A)≥n−k, then for any two rows π and σ of A, there is a position *i* such that |π(i)−σ(i)|≥n−k. So, one of π(i) or σ(i) is ≥n−k+1 and the other must be ≤k. Consequently, one of π(i) or σ(i) is transformed into a 2 and the other into a 0. So the rows $\pi^{\prime }$ and $\sigma^{\prime }$ are separated in $A^{\prime }$. Furthermore, as any two rows of $A^{\prime }$ are separated, any two such rows must be distinct. Therefore $A^{\prime }$ is an (n,k,k)-array and $R\left(n;k,k\right)\geq |A^{\prime }\left|=|A\right|$, so the inequality follows.

Consider now the second inequality. Let *B* be an (n,k,k)-array with the maximum possible number R(n;k,k) of rows. Let $B^{\prime }$ be the permutation array obtained from *B* by arbitrarily replacing, in any row of *B*, the *k* many 2’s by the high potent symbols n−k+1,n−k+2,…,n, the *k* many 0’s by the low potent symbols 1,2,…,k, and the 1 symbols by the symbols k+1,…,n−k. (It is, of course, required that the replacements create a permutation). The replacements performed on any two rows of *B* are performed independently of each other. Since *B* is separable, given any two rows *r* and *s* of $B^{\prime }$, there is a column *c* in $B^{\prime }$ for which one of r(c), s(c) is a high potent symbol while the other is a low potent symbol. So, we have |r(c)−s(c)|≥n−2k+1. Since rows *r* and *s* were arbitrary, this shows that $d(B^{\prime })\geq n-2k+1$. So, by monotonicity in *d* of P(n,d), we obtain $P\left(n,n-2k+1\right)\geq |B^{\prime }|=|B|=R\left(n,k,k\right)$. □

The following Corollary of Lemma 1 shows that R(n;k,k) reaches a maximum that depends only on *k*.

**Corollary 1.**  *There are constants $n_{k}$ and $m_{k}$ (depending only on k) such that for all $n\geq n_{k}$, we have $R\left(n;k,k\right)\leq m_{k}$. Moreover, we can take $m_{k}=c_{2k-1}$ and $n_{k}=2k+d_{2k-1}-1$. Here, $c_{2k-1}$ and $d_{2k-1}$ are the constants from Theorem 1.*

**Proof.**  By the second inequality of Lemma 1, we have R(n;k,k)≤P(n,n−2k+1)=

P(n−2k+1+(2k−1),n−2k+1). So, by Theorem 1, $P\left(n,n-2k+1\right)\leq c_{2k-1}$ for $n-2k+1\geq d_{2k-1}$; that is, $R\left(n;k,k\right)\leq c_{2k-1}$ for $n\geq 2k+d_{2k-1}-1$. □

We note that a transformation of an (n,k,k)-array with *N* rows into an (n,n−k)-PA with *N* rows is not known to be always possible.

We will see later that the existence of the constants $n_{k}$ and $m_{k}$ follows from one of our theorems (Theorem 6), together with an improvement on the bounds given in Theorem 1 for the constants $c_{r}$ and $d_{r}$. Still, we mention Corollary 1 here to show that the existence of $n_{k}$ and $m_{k}$ is already implied by Theorem 1 combined with the argument in that Corollary.

There are a few other theorems in the literature that give upper bounds on the Chebyshev function. Let V(n,d) be the number of permutations on {1,2,…,n} within Chebyshev distance *d* of the identity permutation.

**Theorem 2**  (Theorem 11 [2])**.** *For even d and 2d≥n≥d≥2, $P\left(n,d\right)\leq \frac{(n+1)!}{V(n+1,d/2)}$.*

**Theorem 3**  (Theorem 12 [1])**.** *For 1≤k≤d<n,*

P(n,d)≤P(n−k,d)·nk.

**Corollary 2.**  *For s≤t and 1≤k≤s,*

R(n;s,t)≤R(n−k;s−k,t)·nk.

**Proof.**  Consider any (n,s,t)-array *A*, which we can take to be of maximum possible size R(n;s,t). Create subsets of the rows of *A*, determined by the positions of their *k* many 0’s. That is, two rows are in the same subset if they both have *k* many 0’s occurring in the same *k* positions. There are nk such sets. Any two rows in such a set must be separated somewhere in the remaining n−k positions, using s−k many 0’s and *t* many 2’s in those n−k positions. Hence, any such set of rows must have the size at most R(n−k;s−k,t). It follows that |R(n;s,t)|=|A|≤R(n−k;s−k,t)·nk. □

**Corollary 3.**  *For all 2≤t≤n−2,R(n;2,t)≤n2.*

**Proof.**  Setting s=k=2 in Corollary 2, we have R(n−2,s−k,t)=R(n−2;0,t)=1 since R(m,0,t)=1 for all *m*. □

We will see later (Theorem 9) a bound on R(2,t) that depends only on *t* once *n* is big enough. But, the bound in Corollary 3 is still the best for *n* that is small enough relative to *t*, as we will see.

Some of the best previous upper bounds for small *n* were given using Theorem 3. For example, from Theorem 3, P(n,n−3)≤min{n3,3n2,10n1}, choosing k=3,2,1, respectively. To see this, consider the following. Since P(r,r)=1 for any *r*, taking k=3, we obtain P(n−3,n−3)=1. As mentioned previously after Theorem 1, $c_{1}=3$, so, taking k=2, we obtain P(n−2,n−3)=3. Again, recalling $c_{2}=10$, we take k=1 to obtain P(n−3,n−1)=10. Since min{n3,3n2,10n1}=10n for all n≥10, Theorem 3 gives the upper bound P(n,n−3)≤10n. In an application of our recursive upper bounds, we will see later in Corollary 7 that R(n;3,3)≤169, yielded by Lemma 1. P(n,n−3)≤R(n;3,3)≤169. Thus, Theorem 3 gave the best upper bound for P(n,n−3)≤10n for n≤16, while our new recursive results give an improved bound for P(n,n−3)≤169 when n≥17.

Similarly, from Theorem 3, P(n,n−4)≤minU, where U={n4,3n3,10n2,P(n,n−3)n1}, choosing k=4,3,2,1, respectively. The previous paragraph shows that P(n,n−3)≤169 for all n≥17. Calculating, minU=10n2 for 14≤n≤34 and =169n for all n≥35. In Corollary 8, which we will see later, we obtain R(n;4,4)≤3087. Calculating, one observes that 3087≤minU for all n≥19. So, we obtain the improved upper bound: P(n,n−4)≤R(n;4,4)≤3087 for all n≥19.

We observe that R(n;a,b) is symmetric and monotone; that is,

(3) $$\begin{array}{cc} R(n;a,b) & =R(n;b,a), \end{array}$$

(4) $$\begin{array}{cc} R(n;a,b) & \geq R(m;a,b)\;\mathrm{if}\;n\geq m. \end{array}$$

Later in this paper, it will be useful to consider separable arrays on {0,1,2} in which the number of 0’s and 2’s in each row is not constant for all rows. The following definition and the lemma which follows treat this case.

**Definition 2.**  *For a,b≥2, an (n,≤a,≤b)-array is a separable array of length n strings over {0,1,2} such that each string in A has at most a many 0’s and at most b many 2’s. Let R(n;≤a,≤b) be the maximum size of any (n,≤a,≤b)-array.*

**Lemma 2.**  *If s,t≥1 and n≥s+t, then*

(5) R(n;≤s,≤t)≤R(n;s,t).

**Proof.**  Let *A* be an (n,≤s,≤t)-array with entries from {0,1,2}, realizing R(n;≤s,≤t). Consider any string π in *A* having $s^{\prime }\leq s$ many 0’s and $t^{\prime }\leq t$ many 2’s. Since n−s−t≥0, we have $n-s^{\prime }-t^{\prime }\geq s-s^{\prime }+t-t^{\prime }$, so *A* must have at least $s-s^{\prime }+t-t^{\prime }$ many 1’s in its row corresponding to π. We transform π into a string $\pi^{\prime }$ with *s* many 0’s and *t* many 2’s as follows. We convert any $s-s^{\prime }$ of the 1’s in π to 0’s and any $t-t^{\prime }$ of the remaining 1’s to 2’s and let $\pi^{\prime }$ be the resulting string. Let $A^{\prime }$ be the array obtained from *A* by replacing each π∈A by $\pi^{\prime }$.

It suffices to show that $A^{\prime }$ is separable. It would then follow that no two strings $\pi^{\prime },\sigma^{\prime }\in A^{\prime }$ are the same, since they would not be separated, and since the number of rows in $A^{\prime }$ is at most R(n;s,t), then, also, $R\left(n;s^{\prime },t^{\prime }\right)\leq R\left(n;s,t\right)$. So, let π,σ be two arbitrary strings of *A* and let $\pi^{\prime },\sigma^{\prime }$ be the respective transformed strings in $A^{\prime }$. There is a column *c* of *A* where {π(c),σ(c)}={0,2} (in either order). Since the transformation affects no symbols in π or σ that are either 0 or 2, it follows that $\pi^{\prime }\left(c\right)=\pi \left(c\right)$ and $\sigma^{\prime }\left(c\right)=\sigma \left(c\right)$ and, hence, $\pi^{\prime }$ and $\sigma^{\prime }$ are separated. Thus, $A^{\prime }$ is separable. □

The following theorem will lead to the exact value R(n;2,2)=10 for n≥5.

**Theorem 4.**  *Suppose that $R(n_{0};k,k)\leq m$ such that*

(6) $$2k\left(m+1\right)<\left(n_{0}+1\right)\left(1+\left\lfloor n_{0}/\left(2k-1\right)\right\rfloor \right).$$

*Then, R(n;k,k)≤m for all $n\geq n_{0}$.*

**Proof.**  Suppose to the contrary that R(n;k,k)≥m+1 for some $n>n_{0}$. Let *n* be the smallest such number. Let $A=\{\pi_{1},\pi_{2},\ldots ,\pi_{m+1}\}$ be an (n,k,k)-array. Let $k_{i}$ denote the number of 0 and 2 symbols in position *i*, taken over all rows of *A*. Let $z=1+\lfloor n_{0}/\left(2k-1\right)\rfloor$, so that $n_{0}\geq \left(z-1\right)\left(2k-1\right)$. We show that $k_{i}\geq z$ for all *i*. Suppose, by symmetry of argument, that $k_{1}\leq z-1$ and (by rearranging the order) only $\pi_{i},\;1\leq i\leq k_{1}$, have 0 or 2 symbols in the first position. By our assumption, all of the first $k_{1}$ rows, and only the first $k_{1}$ rows, have a 0 or 2 symbol in position 1. So, if there are z−1 rows, each adding 2k−1 0 or 2 symbols to some position j>1, the total number of 0 or 2 symbols (other than the one in position 1) is (2k−1)(z−1). Since the number of positions, namely, $n>n_{0}$, is greater than (2k−1)(z−1), by the pigeonhole principle, there is a position j>1 where no $\pi_{i},\;1\leq i\leq k_{1}$ do not has any 0 or 2 symbols. Now, do the following:

- For each row $\pi_{i}$, $1\leq i\leq k_{1}$, exchange the 0 or 2 symbol in position 1 with the symbol in position *j*.
- Delete the symbol in position 1 in all rows.

The result is a separable array of ≥m+1 rows, where each row is a string of length n−1. This contradicts our choice of *n* being the smallest. So, we have $k_{i}\geq z$ for all i.

Note that the total number of 0 and 2 symbols in the (n,k,k)-array *A* is ≥2k(m+1). As $k_{i}\geq z$, for all i, we have $2k\left(m+1\right)\geq nz\geq \left(n_{0}+1\right)\left(1+\left\lfloor n_{0}/\left(2k-1\right)\right\rfloor \right)$, which contradicts inequality (6). So, the (n,k,k)-array *A* with ≥m+1 rows does not exist. □

Theorem 15 was used in [1] to prove that P(n,n−2)=10 for all n≥5. A similar proof shows that R(n;2,2)=10 for all n≥5.

**Corollary 4.**  *For all n≥5, R(n;2,2) = 10.*

**Proof.**  R(n;2,2)≥10 for all 5≤n≤11, by computation. In Theorem 4, set $n_{0}=11,k=2$, and m=10. Then, $z=1+\lfloor n_{0}/\left(2k-1\right)\rfloor =4$ and $2k\left(m+1\right)=44<48=\left(n_{0}+1\right)z$. So, R(n;2,2))≤10 for all n≥11, following Theorem 4.

Therefore, R(n;2,2)=10 for all n≥5. □

An example of a (5,2,2)-array with 10 rows realizing R(5;2,2) is given in Figure 1a.

## 3. Applying a Result of Bollobás

We begin with the following result of Bollobás from the theory of extremal sets. It is actually a reformulation, given in [15], of a theorem on saturated hypergraphs originally appearing in [16]. The proof can be found in [15].

**Theorem 5.**  *For two nonnegative integers a and b, write w(a,b)=a+ba−1. Let $\{\left(A_{i},B_{i}:i\in I\right\}$ be a finite collection of finite sets such that $A_{i}\cap B_{j}=\emptyset$ if i=j. For i∈I, set $a_{i}=\left|A_{i}\right|$, $b_{i}=\left|B_{i}\right|$. Then, $\sum_{i\in I}w\left(a_{i},b_{i}\right)\leq 1$ with equality if there is a set Y and integers a,b such that 0≤a≤a+b≤|Y|, and $\left\{\left(A_{i},B_{i}\right):i\in I\right\}$ is the collection of all ordered pairs of subsets of Y with $|A_{i}|=a_{i}$ and $|B_{i}|=b_{i}$.*

*In particular, if $a_{i}=a$ and $b_{i}=b$ for all i∈I, then |I|≤a+ba.*

We obtain an upper bound for R(n;s,t) by reducing to the above theorem.

**Theorem 6.**  *R(n;s,t)≤2s+2ts+t.*

**Proof.**  Let *M* be a an (n,s,t)-array realizing R(n;s,t), and set p=R(n;s,t). For any 1≤i≤p, let $S_{i}=\left\{a_{i1}<a_{i2}<\ldots <a_{is}\right\}$ be the set of column indices at which row *i* of *M* has 0 entries and $T_{i}=\left\{b_{i1}<b_{i2}<\ldots <b_{it}\right\}$ the set of column indices at which row *i* of *M* has 2 entries.

Now, construct a p×2n array *Q* whose first *n* columns are the same as in *M* and whose subarray $M^{\prime }$ consisting of the last *n* columns is obtained by interchanging 0 and 2 entries in *M*, leaving the 1 entries unchanged. That is, $M^{\prime }$ is obtained from *M* by flipping each 2 entry of *M* to a 0 entry in $M^{\prime }$, flipping each 0 entry in *M* to a 2 entry in $M^{\prime }$ and leaving each 1 in *M* unchanged as a 1 entry in $M^{\prime }$. Let $S_{i}^{\prime }=\left\{a_{ij}^{\prime }:1\leq j\leq s\right\}$ (resp. $T_{i}^{\prime }=\left\{b_{ij}^{\prime }:1\leq j\leq t\right\}$) be the column indices in $M^{\prime }$ corresponding to the column indices of $S_{i}$ (resp. $T_{i}$) by a translation of *n*. That is, we have $a_{ij}^{\prime }=a_{ij}+n$ and $b_{ij}^{\prime }=b_{ij}+n$. Now, for each *i*, 1≤i≤p, define two sets of column indices, $Q_{i}$ and $Q_{i}^{\prime }$, of *Q* by $Q_{i}=S_{i}\cup T_{i}^{\prime }$ and $Q_{i}^{\prime }=S_{i}^{\prime }\cup T_{i}$. Observe that $Q_{i}$ (resp. $Q_{i}^{\prime }$) is the set of column indices at which row *i* of *Q* has 0 (resp. 2) entries.

We now show that for 1≤i≤p, the sets $Q_{i}$, $Q_{i}^{\prime }$ can play the roles of $A_{i}$ and $B_{i}$ (respectively) in the statement of Theorem 5 with p=|I|. Trivially, we have $Q_{i}\cap Q_{i}^{\prime }=\emptyset$ for each *i* since $S_{i}\cap T_{i}=\emptyset$. Now, take j≠i, 1≤j≤p. We must show that $Q_{i}\cap Q_{j}^{\prime }\neq \emptyset$ and $Q_{i}^{\prime }\cap Q_{j}\neq \emptyset$. Since *M* is separable, we have $S_{i}\cap T_{j}\neq \emptyset$ or $S_{j}\cap T_{i}\neq \emptyset$, so assume by symmetry that $S_{i}\cap T_{j}\neq \emptyset$. Then, immediately, we have $Q_{i}\cap Q_{j}^{\prime }\neq \emptyset$ since $S_{i}\subset Q_{i}$ and $T_{j}\subset Q_{j}^{\prime }$. Also, it follows from $S_{i}\cap T_{j}\neq \emptyset$ and the interchange of 0’s and 2’s that $S_{i}^{\prime }\cap T_{j}^{\prime }\neq \emptyset$. Therefore, $Q_{i}^{\prime }\cap Q_{j}\neq \emptyset$ since $S_{i}^{\prime }\subset Q_{i}^{\prime }$ and $T_{j}^{\prime }\subset Q_{j}$. Thus, the conditions of Theorem 5 are satisfied. Since $|Q_{i}\left|=\right|Q_{i}^{\prime }|=s+t$ for all *i*, we obtain p=|I|≤2s+2ts+t. □

The preceding theorem implies Corollary 1 with the considerably improved value mk=4k2k over that obtained by combining that corollary and Theorem 1.

**Corollary 5.**  *P(d+r,d)≤4r2r=24r2πr(1+o(1)) (as r grows).*

**Proof.**  By Lemma 1 and Theorem 6, we have P(d+r,d)≤R(d+r;r,r)≤4r2r. The final equality follows from the Stirling approximation applied to 4r2r. □

We note that the preceding corollary implies that $c_{r}\leq \frac{2^{4r}}{\sqrt{2\pi r}}\left(1+o\left(1\right)\right)$, where $c_{r}$ is the constant in Theorem 1. This is an improvement on the upper bound for $c_{r}$ given in that theorem.

## 4. Recursive Techniques

### 4.1. The Positions Method

In this subsection, we introduce a technique, called *positions*, to recursively obtain an upper bound for R(n;a,b). The strategy involves considering a fixed row π of a (n,a,b)-array *A*, with *a* occurrences of the symbol 0 in positions $p_{1},p_{2},\ldots ,p_{a}$ and *b* occurrences of the symbol 2 in positions $q_{1},q_{2},\ldots ,q_{b}$. By separability, every row in *A* other than π must either have a symbol 2 in at least one of the positions $p_{1},p_{2},\ldots ,p_{a}$ or a symbol 0 in at least one of the positions $q_{1},q_{2},\ldots ,q_{b}$. Let $S(p_{i})$ (respectively, $S(q_{i})$) be the set of rows in A with the symbol 2 (respectively, symbol 0) in position $p_{i}$ (resp., $q_{i}$). Each $S(p_{i})$ (resp. $S(q_{i}$) must be a separable subarray of *A*, with separations occurring at positions other than $p_{i}$ (respectively, $q_{i}$). As one 2 (one 0) is used in position $p_{i}$ (resp., $q_{i}$), there are at most *a* many 0’s and at most b−1 many 2’s (resp., a−1 many 0’s and *b* many 2’s) that can be used to separate $S(p_{i})$ (resp. $S(q_{i})$). This method gives the following recursive bound on R(n;a,b).

**Theorem 7.**  *For all a,b≥1 and n≥a+b, R(n;a,b)≤1+aR(n−1;a,b−1)+bR(n−1;a−1,b).*

**Proof.**  Let *A* be an (n,a,b)-array of size R(n;a,b). Let π be a permutation in *A*. Suppose that in π, the 0’s are at positions $p_{1},\ldots ,p_{a}$ and the 2’s are at positions $q_{1},\ldots ,q_{b}$. Every permutation σ∈A−π has a symbol 2 in at least one of the positions $p_{i},1\leq i\leq a$ or a symbol 0 in at least one of the positions $q_{j},1\leq j\leq b$. For every position $p_{i}$, there are at most R(n−1;a,b−1) strings σ∈A−π with $\sigma (p_{i})=2$ and, hence, a total of at most aR(n−1;a,b−1) such strings over all $p_{i}$. For every position $q_{j}$, there are at most R(n−1;a−1,b) strings in σ∈A−π with $\sigma (q_{j})=0$ and, hence, a total of at most bR(n−1;a−1,b) such strings over all $q_{i}$. The bound follows. □

We can obtain an exact formula for R(n;1,k) in the next lemma and the theorem that follows.

**Lemma 3.**  *For all k≥1 and n≥k+1, $R\left(n;1,k\right)\geq \left\{\begin{array}{cc} n & \mathrm{if}\;k+1\leq n\leq 2k,\\ 2k+1 & \mathrm{if}\;n\geq 2k+1. \end{array}\right.$*

**Proof.**  Suppose k+1≤n≤2k+1. Let $\pi_{0}$ be the permutation

$$\pi_{0}=\left(0,\underset{k\;\mathrm{times}}{\underbrace{1,1,\ldots ,1}},\underset{n-k-1\;\mathrm{times}}{\underbrace{2,2,\ldots ,2}}\right).$$

Consider permutations $\pi_{0},\pi_{1},\ldots ,\pi_{n-1}$ defined by $\pi_{i}\left(j\right)=\pi_{0}\left(j-i\right)\;\left(\mathrm{mod}\;n\right)$, that is, $\pi_{i}$ is obtained from $\pi_{0}$ by shifting elements rightward by *i* with wraparound.

First, we observe that the array *A* with rows $\pi_{0},\pi_{1},\ldots ,\pi_{n-1}$, appearing in *A* in order of their index, is separated. It suffices to show that row $\pi_{0}$ is separated from any row $\pi_{i}$, i≥1. The 0 in position 1 of $\pi_{0}$ separates $\pi_{0}$ from the 2 in column 1 of $\pi_{i}$ for 1≤i≤k. The 2’s in columns k+2 through *n* of $\pi_{0}$ each separate from the 0 in the same columns for $\pi_{i}$, k+1≤i≤n−1. Therefore, the permutations $\pi_{i},0\leq i<n$ are pairwise separable and R(n;1,k)≥n.

If n≥2k+1, then R(n;1,k)≥R(2k+1;1,k)≥2k+1, the first inequality by monotonicity of R(n;1,k) for a fixed *k* (see Equation (4)) and the second by the same circular shift construction just given. □

**Theorem 8.** 

*(a)*  *For all k≥1 and n≥k+1, $R\left(n;1,k\right)=\left\{\begin{array}{cc} n & \mathrm{if}\;k+1\leq n\leq 2k,\\ 2k+1 & \mathrm{if}\;n\geq 2k+1. \end{array}\right.$*
*(b)*  *Suppose a separated array A has at most one 0 and at most k many 2’s in each row. If A has 2k+1 rows, then A must have exactly one zero and k many 2’s in each row.*
*(c)*  *Suppose A is an (n,1,k)-array. If A has 2k+1 rows, then A is a (2k+1)×(2k+1) array also with one 0 and k many 2’s in each column.*

**Proof.**  Consider first (a). In view of the lower bound in Lemma 3, it remains only to prove the corresponding upper bounds.

The upper bound R(n;1,k)≤n follows from the fact that any two rows do not have 0 in the same position. Suppose n≥2k+1.

Take an (n,1,k)-array with *p* rows. There are p2=(p−1)p/2 pairs of rows that have to be separated. Let Q be the total number of unordered pairs {0,2} with both the 0 and the 2 lying in the same column of *A*. Then, p2≤Q.

But, Q ≤ {the number of 2’s that are members of such a pair (since there is only one 0 per column)} ≤ {the total number of 2’s in the array}  = pk.

So, we obtain p2≤pk. Solving for *p*, we obtain p≤2k+1.

Consider now (b). Recall that no two 0’s of *A* can be in the same column, since, otherwise, the two rows containing those 0’s cannot be separated. For any column *i* containing a 0, let $s_{i}$ be the number of of 2’s in column *i*. Since *A* is separated, the number of pairwise row separations in *A* is at least 2k+12=k(2k+1). Since there is at most one 0 in each column, we have $\sum_{i}s_{i}\geq k\left(2k+1\right)$.

Assume to the contrary that claim b) is false, so that either some row contains no 0 or some row has fewer than *k* many 0’s. Suppose first that some row contains no 0. Then, since it has at most *k* many 2’s, this row can be separated from most *k* other rows since its separation from other rows can only occur at columns containing its 2’s, and each column has at most one 0. This contradicts *A* being separated, which requires each row to be separated from 2k other rows.

So we may suppose that *A* has 2k+1 many 0’s, but that some row has at most k−1 many 2’s. Then, by the assumption in b), the total number of 2’s in *A* is less than (2k+1)k but is also equal to $\sum_{i}s_{i}$. Then, we have $\left(2k+1\right)k\leq \sum_{i}s_{i}<\left(2k+1\right)k$, a contradiction.

Consider now (c). Since no two 0’s of *A* can be in the same column, it follows that each column has exactly one 0. It also follows that *A* must be a (2k+1)×(2k+1) array.

Suppose to the contrary that some column *c* of *A* has at most k−1 many 2’s. Consider the row *r* passing through the 0 in column *c*. Row *r* must be separated from each of the 2k other rows of *A*. There are k−1 separation pairs involving row *r* that use the 0 in column *c*. The remaining k+1 separation pairs involving row *r* must use the *k* many 2’s in row *r*. But each such 2 participates in only a single separation, that being with the unique 0 in its column. Hence, we cannot find k+1 separations involving these 2’s, a contradiction. □

An example of a (7,1,3)-array realizing R(n;1,3)=7 is given in Figure 1b.

In the next lemma and theorem that follow, we use the positions technique to obtain an upper bound on R(n:2,k).

As a notation, for any subarray *B* of an array *A*, let col(B) (resp, row(B)) be the set of columns (resp. rows) of *B*. Further, let $B^{r}$ (resp. $B^{c}$) be the set of rows (resp. columns) of *A* containing entries of *B*. For a particular column *c*, 1≤c≤n, in some array *A* of *n* columns, we refer to it just by its index *c*. For example, for a subarray *B* of *A*, we write c∩B or B∖c for column(c)∩B or B∖column(c), respectively.

Now, let *A* be an (n,2,k)-array and let *c* be some column of *A*. Let *B* be the subarray consisting of all rows of *A* with a 0 in column *c*. Then, B∖c has one 0 and *k* many 2’s in each row. Assume now that *B* has exactly 2k+1 rows. We then let $\underline{sep(B)}$ be that (n,1,k)-array in *B* (guaranteed to exist by Theorem 8), which has dimensions (2k+1)×(2k+1).

**Lemma 4.**  *Let A be an (n,2,k)-array and let $c_{1},c_{2}$ be two distinct columns of A. Let $S_{1}$ (resp. $S_{2}$) be the subarray of A whose rows have a 0 entry in column $c_{1}$ (resp. $c_{2}$). If $|S_{1}\left|=\right|S_{2}|=2k+1$, then $|S_{1}^{r}\cap S_{2}^{r})|=1$.*

**Proof.**  Since each row of $S_{1}\setminus c_{1}$ and $S_{2}\setminus c_{2}$ has one 0 and *k* many 2’s, we have $|S_{i}|\leq R\left(1,k\right)=2k+1$ by Theorem 8. By our assumption and the same theorem, we then see that $sep(S_{i})$, i=1,2, is a (2k+1,1,k)-array with dimensions (2k+1)×(2k+1) and one 0 and *k* many 2’s in each row and in each column. Note also that if $|S_{1}^{r}\cap S_{2}^{r}|\geq 2$, then any two rows in this intersection have both 0’s in the same two coordinates $c_{1}$ and $c_{2}$ and, hence, cannot be separated, a contradiction to *A* being separated.

We are thus reduced to showing that $|S_{1}^{r}\cap S_{2}^{r}|=0$ leads to a contradiction. Slightly abusing previous notation, in what follows, we use the term *potent symbol* to refer to either a 0 symbol or a 2 symbol in *A*.

Assume that $|S_{1}^{r}\cap S_{2}^{r}|=0$. It follows that every entry in $c_{1}\cap S_{2}$ is nonzero. So, $c_{1}\cap S_{2}\notin col\left(sep\left(S_{2}\right)\right)$ since every column of $sep(S_{2})$ has a 0. Since each row of $sep(S_{2})$ contains one 0 and *k* many 2’s, and since every entry of $c_{2}\cap S_{2}$ is 0 by definition, it follows that every potent symbol in $S_{2}$ lies in $\left(c_{2}\cap S_{2}\right)\cup sep\left(S_{2}\right)$. So, there remain no potent symbols of $S_{2}$ that can appear in $c_{1}\cap S_{2}$. So, every entry in $c_{1}\cap S_{2}$ is 1. By a symmetric argument, we also have that every potent symbol in $S_{1}$ lies in $\left(c_{1}\cap S_{1}\right)\cup sep\left(S_{1}\right)$ and that every entry of $c_{2}\cap S_{1}$ is 1. It follows that all $S_{1}-S_{2}$ cross separations must occur in the columns contained in $sep{\left(S_{1}\right)}^{c}\cap sep\left(S_{2}\right){)}^{c}$.

The number of $S_{1}-S_{2}$ cross separations must be at least ${\left(2k+1\right)}^{2}$, since every row of $S_{1}$ must be separated from every row of $S_{2}$. Now, in each column $c\in sep{\left(S_{1}\right)}^{c}\cap sep{\left(S_{2}\right)}^{c}$, there are 2k many $S_{1}-S_{2}$ cross separations, obtained by pairing the 0 in $c\cap sep(S_{1})$ with each of the *k* many 2’s in $c\cap sep(S_{2})$, and the same with $S_{1}$ and $S_{2}$ interchanged. Since $|sep{\left(S_{1}\right)}^{c}\cap sep{\left(S_{2}\right)}^{c}|\leq 2k+1$, the total number of $S_{1}-S_{2}$ cross separations is at most $2k\left(2k+1\right)<{\left(2k+1\right)}^{2}$, a contradiction. □

In the theorem that follows, we abbreviate the symbols R(n;2,k), R(n−1;2,k−1), and so on by R(2,k) or R(2,k−1); that is, we drop the first coordinate in the *R* function. We take *n* large enough so that R(n;2,k) depends only on *k* (see Corollary 1). By monotonicity, the upper bound we then obtain for R(n,2,k) holds also for $R(n^{\prime },2,k)$, where $n^{\prime }<n$.

**Theorem 9.**  *$R\left(2,k\right)\leq \frac{k(k+4)(2k+1)}{3}-10$.*

**Proof.**  Let *A* be be an (n,2,k) array achieving the maximum possible number of rows R(2,k) for such arrays. Let π be a fixed row of *A*, with its two 0’s in columns $p_{1}$ and $p_{2}$ and its *k* many 2’s in columns $q_{1},q_{2},\ldots ,q_{k}$. Every row of A∖π, being separable from π, must have a 2 in at least one of the columns $p_{1}$ and $p_{2}$ or a 0 in at least one of the columns $q_{1},q_{2},\ldots ,q_{k}$.

Let $T_{1}$ (resp. $T_{2}$) be the subarray of A∖π consisting of the rows of *A* with a 2 in column $p_{1}$ (resp. $p_{2}$). Note that any row of $T_{1}\cup T_{2}$ has at most k−1 many 2’s outside the columns $p_{1},p_{2}.$

First, we give an upper bound for $|T_{1}\cup T_{2}|$ as follows. Let $B_{1}$ be the set of rows in $T_{1}\cup T_{2}$ with no 0 entry in columns $p_{1},p_{2}$. Then, $|B_{1}|\leq R\left(2,\leq k-1\right)\leq R\left(2,k-1\right)$ by Lemma 2. Let $B_{2}$ be the set of rows in $T_{1}\cup T_{2}$ with exactly one 0 in one of the columns $p_{1}$ or $p_{2}$. Then, by Lemma 4 and Theorem 8, we have $|B_{2}|\leq 2R\left(1,k-1\right)-1=4k-3$. Finally, no row of $T_{1}\cup T_{2}$ can have both its 0’s in columns $p_{1},p_{2}$ since such a row would not be separated from π. Therefore, we have $|T_{1}\cup T_{2}\left|\leq \right|B_{1}\left|+\right|B_{2}|\leq R\left(2,k-1\right)+4k-3$.

Let $S_{i},1\leq i\leq k$, be the subarray of *A* consisting of the rows of *A* with a 0 in column $q_{i},1\leq i\leq k$. Note that $|S_{i}|\leq R\left(1,\leq k\right)\leq R\left(1,k\right)=2k+1$.

We now give an upper bound for $|\cup_{i=1}^{k}S_{i}^{r}|$. First note that for any triple of indices 1≤i<j<t≤k, we have $|S_{i}^{r}\cap S_{j}^{r}\cap S_{t}^{r}|=0$, since any row of *A* contained in this triple intersection has three 0 entries, contradicting *A* being an (n,2,k)-array. Applying inclusion–exclusion, we thus obtain

(7) $$|\cup_{i=1}^{k}S_{i}^{r}|=\sum_{1\leq i\leq k}|S_{i}^{r}|-\sum_{1\leq i<j\leq k}\left|S_{i}^{r}\cap S_{j}^{r}\right|.$$

Also note that $|S_{i}^{r}\cap S_{j}^{r}|=0$ or 1 since any two rows of *A* contained in $S_{i}^{r}\cap S_{j}^{r}$ have both of their 0 entries in the same two columns $q_{i},q_{j}$ and, hence, cannot be separated.

We now maximize the right side of (7) over all possible collections of subarrays $S_{i},1\leq i\leq k$ of *A* as defined above. Let $|S_{i}^{r}|=2k+1$ for 1≤i≤t, while $|S_{i}^{r}|\leq 2k$ for t+1≤i≤k. By Lemma 4, we have $|S_{i}^{r}\cap S_{j}^{r}|=1$ for 1≤i<j≤t. Therefore, we obtain

|∪i=1kSir|≤(2k+1)t+(k−t)2k−t2=g(t).

To maximize g(t) on the domain 1≤r≤k, we differentiate to obtain $g^{\prime }\left(t\right)=\frac{3}{2}-t$, so $t=\frac{3}{2}$ is the only critical point, and, also, $g^{\prime }\left(1\right)>0$, while $g^{\prime }\left(2\right)<0$. So, the maximum of g(t) at integer values 1≤t≤k is max$\left\{g\left(1\right),g\left(2\right)\right\}=2k^{2}+1.$ So, we have $|\cup_{i=1}^{k}S_{i}|\leq 2k^{2}+1$.

Finally, for k≥3, we obtain the following recurrence, where the first summand “1” accounts for the fixed permutation π.

$R\left(2,k\right)\leq 1+|T_{1}\cup T_{2}|+|\cup_{i=1}^{k}S_{i}^{r}|\leq R\left(2,k-1\right)+2k^{2}+4k-1$.

We can unravel this recurrence to obtain

$$\begin{array}{cc} R(2,k) & \leq R\left(2,2\right)+2\sum_{i=3}^{k}i^{2}+4\sum_{i=3}^{k}i-\left(k-2\right)\\ \; & =10+2\left(\frac{k(k+1)(2k+1)}{6}-1^{2}-2^{2}\right)+4\left(\frac{k(k+1)}{2}-1-2\right)-\left(k-2\right)\\ \; & =\frac{k(k+4)(2k+1)}{3}-10. \end{array}$$

□

We note that the upper bound on R(n;2,k) from Theorem 9, being independent of *n* once *n* is big enough, is better than the bound R(n;2,k)≤n2 from Corollary 3 for *n* that is large relative to *k*, but the latter bound is stronger when $n\leq Ck^{3/2}$ for a suitable constant *C*. Also, the bound from Theorem 9 is stronger than the bound R(n;2,k)≤(2k+4k+2) from Theorem 6 for all but small *k*.

As examples to be used later, we mention the following.

**Corollary 6.**  *R(2,3)≤39 and R(2,4)≤86.*

### 4.2. The Partition Method

In this subsection, we develop a recursive method, which we call the *partition method*, which, in some sense, generalizes the positions method of the previous subsection. In the partition method, we consider subarrays of a separable array *A* over {0,1,2} defined by restrictions of rows of *A* to a certain set of coordinates in *A*. In the preceding positions method, the subarrays were defined by their restriction to a single coordinate.

Let *A* be an (n,s,t)-array with, say, s≤t. Choose a row π∈A with *s* occurrences of the symbol 0 in positions $p_{1},\ldots ,p_{s}$ and *t* occurrences of the symbol 2 in positions $q_{1},\ldots ,q_{t}$. For separation, all rows in *A* other than π must have either a symbol 2 in one of the positions $p_{1},\ldots ,p_{s}$ or a symbol 0 in one of the positions $q_{1},\ldots ,q_{t}$. Let *S* be the set of strings in *A* with at least one 2 in the positions $\left\{p_{1},p_{2},\ldots ,p_{s}\right\}$ and let *T* be the set of strings in *A* with at least one 0 in the positions $\left\{q_{1},q_{2},\ldots ,q_{t}\right\}$. Since every string in *A* is separated from π, we have A={π}∪S∪T, so |A|≤1+|S|+|T|. In this section, we upper bound |S| (and similarly |T|) by partitioning *S* into certain collections of strings, and then upper bound the sizes of each of these collections. The collections come in two types as follows. For any string σ∈S, let $\sigma_{P}$ be the length *s* restriction of σ to the positions $\left\{p_{1},p_{2},\ldots ,p_{s}\right\}$; that is, $\sigma_{P}=\sigma_{p_{1}}\sigma_{p_{2}}\ldots \sigma_{p_{s}}$. Also, let $\tau (\sigma_{P})$ be the set of positions among $\left\{p_{1},p_{2},\ldots ,p_{s}\right\}$ at which $\sigma_{P}$ has a 2 symbol. The two types of collections are the following.

(1) $S_{0}=\{\sigma \in S:\sigma_{P}$ has no 0 symbols}.
(2) For each nonempty subset $D\subseteq \{p_{1},p_{2},\ldots ,p_{s}\}$ satisfying |D|≤s−1, let $S_{D}=\{\sigma \in S:\sigma_{P}$ contain at least one 0 and $\tau (\sigma_{P})=D\}$.

Clearly, $S=S_{0}\cup (\underset{D}{⋃}S_{D})$ is a partition of *S*, so $|S|=|S_{0}|+\sum_{D}\left|S_{D}\right|$. We upper bound the sizes of these sets of rows in the following lemma.

**Lemma 5.**  *The sets $S_{0}$, $S_{D}$ satisfy the following.*

*(a)*  *No two strings in $S_{0}$ and no two string in $S_{D}$ are separable in any of the coordinates $\left\{p_{i},1\leq i\leq s\right\}$. So, all internal separations in $S_{0}$ and in $S_{D}$ occur outside the coordinates $p_{i},1\leq i\leq s$.*
*(b)*  *$|S_{0}|\leq R\left(n-s,s,\leq t-1\right)\leq R\left(n-s,s,t-1\right)$.*
*(c)*  *$|S_{D}|\leq R(n-s,\leq s-1,t-|D|)\leq R(n-s,s-1,t-|D|)$.*

**Proof.**  For (a), no two strings in $S_{0}$ are separable in one of the coordinates $\left\{p_{i},1\leq i\leq s\right\}$, since neither has a 0 in those coordinates. Similarly, no two strings σ, γ in any $S_{D}$ are separable in a coordinate $c\in \{p_{i},1\leq i\leq s\}$ since we have $\sigma_{c}=2$ if and only if $\gamma_{c}=2$. Thus, all internal separations in $S_{0}$ or in any $S_{D}$ occur in columns outside $p_{1},p_{2},\ldots ,p_{s}$. We then define the subarrays $S_{0}^{\prime }$ and $S_{D}^{\prime }$ of *A* by

$$S_{0}^{\prime }=\left\{\sigma \setminus \sigma_{P}:\sigma \in S_{0}\right\}\;\mathrm{and}\;S_{D}^{\prime }=\left\{\sigma \setminus \sigma_{P}:\sigma \in S_{D}\right\}.$$

So, $S_{0}^{\prime }$ (resp. $S_{D}^{\prime }$) is the set of length n−s strings obtained by deleting the substring $\sigma_{P}$ from each string $\sigma \in S_{0}$ (resp. $\sigma \in S_{D}$). Note that $|S_{0}|=|S_{0}^{\prime }|$ since for any two strings $\sigma ,\gamma \in S_{0}$, we have $\sigma \setminus \sigma_{P}\neq \gamma \setminus \gamma_{P}$ because, for some coordinate *c* outside $p_{1},p_{2},\ldots ,p_{s}$, we must have $\sigma_{c}=2$ and $\gamma_{c}=0$. This is because all internal separation in $S_{0}$ occurs outside the $p_{i}$ coordinates, as observed above. Similarly, $|S_{D}|=|S_{D}^{\prime }|$ for any nonempty subset $D\subseteq \{p_{1},p_{2},\ldots ,p_{s}\}$.

Consider now part (b). Since any $\sigma \in S_{0}$ has no 0’s in positions $p_{i},1\leq i\leq s$, then $\sigma \setminus \sigma_{P}$ is a length n−s string containing *s* many 0’s and at most t−1 many 2’s. Hence, $|S_{0}|=|S_{0}^{\prime }|\leq R\left(n-s,s,\leq t-1\right)$. The second inequality then follows Lemma 2.

For part (c), note that by definition for any $\gamma \in S_{D}$, $\gamma_{P}$ contains at least one 0 and |D| many 2’s. So, $\gamma \setminus \gamma_{P}$ is a length n−s string that has at most s−1 many 0’s and at most t−|D| many 2’s. So, we obtain $|S_{D}|=|S_{D^{\prime }}|\leq R(n-s,\leq s-1,t-|D|)$. Again, the second inequality follows Lemma 2. □

We mention the analogue of Lemma 5 for subsets of *T* that correspond to $S_{0}$ and the sets $S_{D}$. For any string σ∈T, let $\sigma_{Q}$ be the length *t* restriction of σ to the positions $\left\{q_{1},q_{2},\ldots ,q_{t}\right\}$; that is, $\sigma_{Q}=\sigma_{q_{1}}\sigma_{q_{2}}\ldots \sigma_{q_{t}}$. Also, let $\tau^{\prime }\left(\sigma_{Q}\right)$ be the set of positions among $\left\{q_{1},q_{2},\ldots ,q_{t}\right\}$ at which $\sigma_{Q}$ has a 0 symbol. In a similar way, one can define sets of rows $T_{0}$ and $T_{E}$ within *T* as follows.

(1) $T_{2}=\{\sigma \in T:\sigma_{Q}$ has no 2 symbols}.
(2) For each nonempty subset $E\subseteq \{q_{1},q_{2},\ldots ,q_{t}\}$, |E|≤s, let $T_{E}=\{\sigma \in T:\sigma_{Q}$ contain at least one 2 and $\tau^{\prime }\left(\sigma_{Q}\right)=E\}$. The restriction |E|≤s is necessary since each row in *A* has at most *s* many 0’s.

Again, we have $T=T_{2}\cup (\underset{E}{⋃}T_{E})$ as a partition of *T*, so $|T|=|T_{2}|+\sum_{E}\left|T_{E}\right|$. The corresponding upper bounds for $|T_{2}|$ and $|T_{E}|$ are given in the following lemma. We omit the proof as it is entirely analogous to the proof of Lemma 5.

**Lemma 6.**  *The sets $T_{2}$ and $T_{E}$ satisfy the following.*

*(a)*  *No two strings in $T_{0}$ and no two strings in $T_{E}$ are separable in the coordinates $q_{i},1\leq i\leq s$. So, all internal separations in $T_{0}$ and in $T_{E}$ occur outside the coordinates $q_{i},1\leq i\leq t$.*
*(b)* 
$|T_{2}|\leq R\left(n-t,\leq s-1,t\right)\leq R\left(n-t,s-1,t\right)$

*(c)*  *$|T_{E}|\leq R(n-t,\leq s-|E|,\leq t-1)\leq R(n-t,s-|E|,t-1)$.*

We illustrate the use of the partition method for upper bounding R(n;3,3) in the following corollary.

**Corollary 7.**  *R(n;3,3)≤169.*

**Proof.**  Consider an (n,3,3) array *A* achieving R(n;3,3). We find the sets $S_{0}$, $S_{D}$ (with a symmetric procedure for finding the sets $T_{2},T_{E}$). Then, we use Lemma 5 and other theorems to upper bound $|S_{0}|$ and $|S_{D}|$ for each $D\subset \{p_{1},p_{2},p_{3}\}$, |D|≤2. From this, we obtain a bound for *S* and, using Lemma 6, a symmetric bound on *T*. Finally, using |A|≤1+|S|+|T|, we obtain our bound for R(n;3,3).

Again, we take π to a row of an (n,3,3) array *A*, with its three 0’s in coordinates $p_{1}<p_{2}<p_{3}$ and its three 2’s in coordinates $q_{1}<q_{2}<q_{3}$. We describe the sets of rows in $S_{0}$ or $S_{D}$ by specifying for each row σ in such a set its length 3 restriction $\sigma_{P}$ to $p_{1},p_{2},p_{3}$. Then, we upper bound $S_{0}$ and $S_{D}$ using the preceding lemmas and additional results already given. The justification for these bounds are given after the list of sets $S_{0}$ and $S_{D}$.

1. $S_{0}=\left\{\sigma \in S:\sigma_{P}\in \left\{222,221,212,122,211,121,112\right\}\right\}$, $|S_{0}|\leq R\left(n-3,3,\leq 2\right)\leq 39$.
2. $D_{1}=\left\{p_{1},p_{2}\right\}$, $S_{D_{1}}=\left\{\sigma \in S:\sigma_{P}=\left\{220\right\}\right\}$, $|S_{D_{1}}|\leq R\left(n-3,2,1\right)\leq 5$.
3. $D_{2}=\left\{p_{1},p_{3}\right\}$, $S_{D_{2}}=\left\{\sigma \in S:\sigma_{P}=\left\{202\right\}\right\}$, $|S_{D_{2}}|\leq R\left(n-3,2,1\right)\leq 5$.
4. $D_{3}=\left\{p_{2},p_{3}\right\}$, $S_{D_{3}}=\left\{\sigma \in S:\sigma_{P}=\left\{022\right\}\right\}$, $|S_{D_{3}}|\leq R\left(n-3,2,1\right)\leq 5$.
5. $D_{4}=\left\{p_{1}\right\}$, $S_{D_{4}}=\left\{\sigma \in S:\sigma_{P}=\left\{201,210,200\right\}\right\}$, $|S_{D_{4}}|\leq R\left(n-3,2,2\right)\leq 10$.
6. $D_{5}=\left\{p_{2}\right\}$, $S_{D_{5}}=\left\{\sigma \in S:\sigma_{P}=\left\{021,120,020\right\}\right\}$, $|S_{D_{5}}|\leq R\left(n-3,2,2\right)\leq 10$.
7. $D_{6}=\left\{p_{3}\right\}$, $S_{D_{6}}=\left\{\sigma \in S:\sigma_{P}=\left\{012,102,002\right\}\right\}$, $|S_{D_{6}}|\leq R\left(n-3,2,2\right)\leq 10$.

The bound for $S_{0}$ in item 1 comes from Theorem 9, for $S_{D_{i}}$ in items 2–4 from Theorem 8, and in items 5–7 from Corollary 4. We obtain $|S|=|S_{0}|+\sum_{D}\left|S_{D}\right|=84$ by symmetry |T|=84 using sets $T_{2}$ and $T_{E}$, as in Lemma 6. Finally, we have R(n;3,3)=|A|≤1+|S|+|T|≤169. □

Note that from Lemma 1, we then have P(d+3,d)≤169, an improvement over the previous bound $P\left(d+3,d\right)\leq 2^{6}\left(6!\right)=46,080$, cited in Theorem 1. This bound is also an improvement on the bound P(d+3,d)≤126=924 in Corollary 5 derived from the theorem of Bollobás.

The partition technique shown in the above example is generalized in the next two theorems.

**Theorem 10.**  *For all k≥3, R(n;k,k)≤1+2∑i=0k−1ki·R(n−k;k−1,k−i).*

**Proof.**  Let *A* be an (n,k,k) array realizing R(n;k,k) and let π be a row of *A*. As usual, we take π to have 0’s in positions $p_{1},p_{2},\ldots ,p_{k}$ and 2’s in positions $q_{1},q_{2},\ldots ,q_{k}$. We continue with the notation $S,T,S_{0},S_{D},T_{0},T_{E}$ from the two lemmas preceding this theorem and we take s=t=k in those lemmas. In particular, we have |A|=1+|S|+|T|, and we now proceed to estimate |S|, the estimate for |T| being identical by symmetry.

By Lemma 5, we have $S_{0}\leq R\left(n-k;k,k-1\right)$.

For each subset $D\subseteq \{p_{1},p_{2},\ldots ,p_{k}\}$, we have by Lemma 5 that $|S_{D}|\leq R(n-k,k-1,k-|D|)$. If |D|=i, there are ki such *D*’s. Since $|S|=|S_{0}|+\sum_{D}\left|S_{D}\right|$, we obtain

(8) |S|≤R(n−k;k,k−1)+∑i=1k−1ki·R(n−k;≤k−1,k−i),so

(9) |S|≤∑i=0k−1ki·R(n−k;≤k−1,k−i),

We have the same bound for *T* based on Lemma 6 and $|T|=|T_{0}|+\sum_{E}\left|T_{E}\right|$. Since |A|=1+|S|+|T|, we then obtain

(10) R(n;k,k)≤1+2∑i=0k−1ki·R(n−k;≤k−1,k−i).

By Lemma 2 and Equation (4), the theorem follows. □

**Theorem 11.**  *For all t>s≥2,*

R(n;s,t)≤1+R(n−s;s,t−1)+R(n−t;s−1,t) +∑i=1s−1si·R(n−s;s−1,t−i)+∑i=1sti·R(n−t;s−i,t−1).

**Proof.**  We continue with the notation of Theorem 10 and the Lemmas that precede it.

Using exactly the same reasoning as in Theorem 10, we obtain

|S|≤R(n−s;s,t−1)+∑i=1s−1si·R(n−s;s−1,t−i).

The estimate for *T* is very similar, except for a restriction on the sizes of sets *E* defining the sets $T_{E}$.

By Lemma 6, we have $T_{2}\leq R\left(n-t,s-1,t\right)$. By the same lemma, we have that for any $E\subset \{q_{1},q_{2},\ldots ,q_{t}\}$ with the size restriction |E|≤s, we have $|T_{E}|\leq R(n-t,s-|E|,t-1)$. Since there are ti, 1≤i≤s choices for the set *E*, we obtain

(11) |T|≤R(n−t;s−1,t)+∑i=1sti·R(n−t;s−i,t−1).

Finally, the theorem follows from Equations (11) and the preceding bound for |S|. □

We now calculate some values from the above recurrences.

**Corollary 8.**  *R(n;3,4)≤605,R(n;4,4)≤3,087,R(n;3,5)≤1,669,R(n;4,5)≤12,327, and R(n;5,5)≤69,435.*

**Proof.**  We denote R(n;s,t) by R(s,t) for short (using monotonicity of R(n;s,t) in *n*). Then, the bounds in Theorems 10 and 11 can be written as

R(k,k)≤1+2∑i=0k−1ki·R(k−1,k−i)R(s,t)≤1+∑i=0s−1si·R(s−1,t−i)+∑i=0sti·R(s−i,t−1)=1+(s+t)R(s−1,t−1)+∑1≠i=0s−1si·R(s−1,t−i)+∑1≠i=0sti·R(s−i,t−1).

For starting values in these recurrences, we use Theorem 9 for R(2,3)≤39 and R(2,4)≤86, Corollary 7 for R(3,3)≤169, Theorem 8 for R(3,1)=7, Corollary 4 for R(2,2)=10, and R(0,k)=1 for all *k*. Now, applying the recurrences, we obtain the following values.

1. R(3,4)≤1+7R(2,3)+R(2,4)+3R(2,2)+R(3,3)+6R(1,3)+4R(0,3)≤1+7·39+86+3·10+169+6·7+4·1=605.
2. R(4,4)≤1+2(R(3,4)+4R(3,3)+6R(3,2)+4R(3,1))≤1+2(605+4·169+6·39+4·7) = 3087.
3. R(3,5)≤1+8R(2,4)+R(2,5)+3R(2,3)+R(3,4)+10R(1,4)+10R(0,4)≤1+8·86+158+3·39+605+10·9+10 = 1669.
4. R(4,5)≤1+9R(3,4)+(R(3,5)+6R(3,3)+4R(3,2))+(R(4,4)+10R(2,4)+10R(1,4)+5R(0,4))≤1+9·605+1669+6·169+4·39+3087+10·86+10·9+5 = 12,327.
5. R(5,5)≤1+2(R(4,5)+5R(4,4)+10R(4,3)+10R(4,2)+5R(4,1))≤1+2(12327+5·3087+10·605+10·86+5·9) = 69,435.

□

By Lemma 1, we have P(n,n−4)≤ 3087, so in the notation of Theorem 1, we have $c_{4}\leq 3087$. This is an improvement over that given in inequality (1), namely, $c_{4}\leq 2^{8}\left(8!\right)=10,321,920$. It is also an improvement on the bound P(n,n−4)≤168=12,870 derived from Corollary 5 based on the reduction from the theorem of Bollobás. The latter bound is still best though for large *r*.

Similarly, from the bound R(5,5)≤ 69,435, we obtain $c_{5}\leq$ 69,435. This improves considerably the bound $c_{5}\leq 2^{10}\left(10!\right)$, which is roughly $3.6\times 10^{9}$.

A rough upper bound for R(k,k) obtained by applying the positions technique is $R\left(k,k\right)\leq k^{k-1}(\frac{e}{2}{)}^{k}$. Since $c_{k}\leq R\left(k,k\right)$ (for *n* large enough), this is also a considerable improvement on the bound for $c_{k}$ from inequality (1). The positions and partition techniques give good bounds for R(k,k) (and, hence, $c_{k}$) for moderately large *k*, but, still, the best such bounds so far for large *k* come from Corollary 5. 

## Data Availability

The original contributions presented in this study are included in the article. Further inquiries can be directed to the corresponding author.
